# Paranasal Sinus Hypoplasia and Sinonasal Anatomical Variants in Cystic Fibrosis Adult Patients: A Computed- Tomography-Based Volumetric Comparison with Healthy Controls

**DOI:** 10.3390/jcm14092977

**Published:** 2025-04-25

**Authors:** Camilla Russo, Antonella Miriam Di Lullo, Lorenzo Ugga, Michele Cavaliere, Mario Tortora, Renato Cuocolo, Gisella Nele, Ferdinando Caranci, Francesco Briganti, Giuseppe Castaldo, Andrea Elefante

**Affiliations:** 1 Department of Advanced Biomedical Sciences, University of Naples “Federico II”, Via Sergio Pansini 5, 80131 Naples, Italy; camilla_russo@hotmail.it (C.R.); frabriga@unina.it (F.B.); aelefant@unina.it (A.E.); 2 CEINGE-Advanced Biotechnology, 80131 Naples, Italy; antonella.dilullo@libero.it (A.M.D.L.); giuseppe.castaldo@unina.it (G.C.); 3 Otorhinolaryngology, San Pio Hospital, Via Pacevecchia 53, 82100 Benevento, Italy; 4 Department of Advanced Medical and Surgical Sciences, School of Medical Sciences, University of Campania “Luigi Vanvitelli”, P.zza L. Miraglia 2, 80138 Naples, Italy; lorenzo.ugga@unicampania.it (L.U.); ferdinando.caranci@unicampania.it (F.C.); 5 Division of Otolaryngology-Head and Neck Surgery, Department of Neuroscience, Reproductive and Odontostomatologic Sciences, University of Naples “Federico II”, Via Sergio Pansini 5, 80131 Naples, Italy; michele.cavaliere@unina.it; 6 Department of Medicine, Surgery and Dentistry, University of Salerno, Via Salvator Allende 43, 84081 Baronissi, Italy; rcuocolo@unisa.it; 7 Plastic Surgery “Body&Mind Center”, 80123 Naples, Italy; gisenele@gmail.com; 8 Department of Molecular Medicine and Medical Biotechnology, University of Naples “Federico II”, Via Sergio Pansini 5, 80131 Naples, Italy

**Keywords:** cystic fibrosis, paranasal sinuses, computed tomography, volume assessment

## Abstract

**Objectives:** In this retrospective study, we performed a volumetric analysis of paranasal cavity pneumatization in a population of adult patients with cystic fibrosis compared to healthy controls, providing parcel evaluation of each sinus, and analyzing the prevalence of major anatomical sinonasal variants in the two groups. **Methods:** We compared paranasal sinus volumes of 89 adult patients with cystic fibrosis and 144 healthy controls who underwent paranasal sinus computed tomography. Volumes were segmented and extracted on tomographic images using the freely available software MRIcron 2019, then compared using a t-test; the *z*-score test was used to determine whether the two groups differ significantly in terms of major anatomical variants prevalence. **Results:** Overall sinus volumes in patients with cystic fibrosis patients differ significantly as compared to the healthy population (*p* < 0.00001). Furthermore, with the only exception of ethmoid sinus pneumatization, which was similar in both populations, all the other sinuses were statistically different. No significant difference emerged concerning anatomical variants’ prevalence. **Conclusions:** Our results further stress the impact of cystic fibrosis on sinus structure in adult patients, better revealing the consequences of the disease on upper airways and in optimizing the management of patients with sinonasal manifestations.

## 1. Introduction

Cystic fibrosis (CF) is the most common autosomal recessive disease among Caucasians with a reported incidence of 1:2000 [1,2]. This life-shortening disorder is due to a mutation in the CF transmembrane conductance regulator (CFTR) gene on chromosome 7, which results in ion transporter dysfunction in epithelial cell membranes [3]. Ion transporter protein deficiency leads to high concentrations of ions such as chloride and sodium and mucins, determining the production of thick and viscous exocrine secretions and a global impairment of mucociliary clearance [4]. Clinical manifestations are heterogeneous, with variable involvement of respiratory, digestive, and reproductive systems [5]. Genotype–phenotype correlation in CF is a complex open system, with more convincing evidence collected for pancreatic and lung involvement [6,7,8]; at present, genotype–phenotype correlation for sinonasal involvement is still inconclusive, also due to the role of modifier genes inherited independently by CFTR [9]. This is at least in part because paranasal sinus involvement is not the most serious manifestation (particularly in children with more severe disease), thus receiving much less attention compared to other organs and systems; conversely, such manifestations become of key importance for disease control and life quality in adult CF patients and in cases of less severe disease [10,11]. Upper airway and paranasal sinus implications are nearly constant in this subset of patients, and paranasal sinus symptoms may be a prominent feature of the underlying disease, especially in cases of milder phenotypes; from a clinical standpoint, upper airway manifestations in adult CF patients mainly include chronic sinusopathy and sinonasal polyposis [12].

Despite sufficient knowledge of the clinical manifestations of paranasal sinus involvement in CF, at present, the impact of causative CF mutations on sinus development and expansion is still poorly understood. Previous experimental results obtained on animal models of CF did not clarify whether impaired pneumatization of paranasal sinuses should be considered a consequence or a significant trigger factor of sinus disease [13,14]. At the same time, several clinical studies in recent years provided computed tomography (CT)-based characterization of paranasal sinuses in CF patients and documented alterations in sinuses pneumatization, with high prevalence of frontal sinus aplasia, maxillary sinus hypoplasia, and sphenoidal sinus hypoplasia [12,15,16,17,18,19,20,21]; in particular, bony sclerosis and mucosal inflammation leading to chronic infections have been called into question as the main cause of poor sinus pneumatization. Therefore, scientific evidence on the topic must be considered still controversial, with more evidence collected in children than in adult patients. Regarding sinus expansion in the CF pediatric population, some studies documented that sinuses in children with CF are smaller than healthy controls (HCs) [3,22], but do not provide a volumetric analysis to assess the significance of the presented observations. A single recent study proposed a CT-based volumetric analysis of sinus expansion, proving on a large cohort of pediatric patients and matched controls how CF disease negatively affected the longitudinal development of the paranasal sinuses by slowing their pneumatization and dilation with age [23]. Conversely, concerning the adult CF population, some authors provided a qualitative evaluation of the impact of CF mutation on sinus pneumatization in adults, but did not provide a volumetric analysis [12] or comparison with HCs [21,24]. With this background, we propose a volumetric analysis of paranasal cavity pneumatization in a population of adult patients diagnosed with CF compared to a comparable group of HCs, providing parcel evaluation of each sinus and evaluating their single contribution to overall sinus volumes; we also performed an ancillary analysis on the prevalence of major anatomical sinonasal variants in the two groups.

## 2. Materials and Methods

### 2.1. Participants Selection

In this retrospective study, we considered 138 CF adult patients referred to the Otolaryngology Unit of the University of Naples “Federico II” who underwent paranasal sinus CT examination for clinical suspicion of acute/chronic pansinusitis or sinonasal polyps, thus being potential candidates for endoscopic exploration or surgical procedure. Exclusion criteria were as follows: age < 18 y at the moment of CT examination; no positive chloride sweat test with confirmatory genotyping available; lacking evaluation of sinonasal diseases by means of CT scan; extensive bony erosion or demineralization on CT images, evaluated by an experienced neuroradiologist; sinonasal pathology other than acute/chronic pansinusitis or sinonasal polyps; previous local surgery. CT examinations were carried out in different diagnostic centers, so paranasal sinus CT protocol minimum requirements for patient inclusion were settled as follows: minimum number of detector elements of 16; scan extent from hard palate to above the end of the frontal sinuses; maximum slice thickness of 1.0 mm; possibility to perform multiplanar reconstructions; reconstruction using bone (high frequency) kernels (i.e., ≥ 4000 HU); availability of non-contrast imaging. Of all considered patients, 49 individuals were excluded due to low image quality not adherent to CT minimum requirements (n = 13), motion artifacts (n = 5), metal artifacts caused by dental implants (n = 12), impossibility of retrieving CT images (n = 2), incomplete medical records with neither sweat test nor confirmatory genotyping (n = 3), extensive bony erosion or demineralization on CT images due to previous trauma, surgical interventions, or complications from dental implant procedures, which have caused a significant alteration of the original anatomy of the paranasal region (n = 14). Finally, we recruited 89 consecutive unrelated CF adult patients (47 males and 42 females; mean age 33.94 ± 10.38 y; age range 18–61 y). Concerning the included CF patients, confirmatory genotyping after a positive chloride sweat test documented class I–II–III mutations (such as deltaF508, N1303K, G542X) in 80% of cases, and class IV–V–VI mutations in the remaining 20% of cases, according to the Italian Cystic Fibrosis Registry Report; class I–II–III mutations are often associated with a more severe phenotype, while class IV–V–VI mutations are generally linked to milder manifestations. Inclusion was therefore limited to patients with high-quality imaging and complete clinical records, ensuring data reliability and reproducibility. We then retrospectively selected 144 age- and sex-comparable healthy controls (86 males and 58 females; mean age 27.60 ± 8.51 y; age range 18–59 y; ratio CF:HC of about 1:1.5) who underwent paranasal sinus CT examination (with the same CT protocol minimum requirement criteria) for aesthetic plastic surgery planning (n = 96), minor facial trauma with no evidence of bone fractures and bone bruises (n = 46), or suspected foreign body retention (n = 2); patients with incidental detection of paranasal sinus abnormalities on imaging, unrelated to the clinical indication for CT (such as mucosal thickening and retention cyst) and with no clinical equivalent, were still included in the analysis. For all subjects, sinonasal anatomical variants [25] detected on CT examination were recorded (i.e., concha bullosa, infraorbital canal dehiscence, sphenoid septum, clinoid pneumatization, Onodi cell, Haller cells, Agger nasi, and Ethmoid bulla) and their prevalence calculated; the Lund–Mackay score for staging chronic rhinosinusitis was also reported. Demographics and anatomical variants of recruited CF and HC subjects are shown in Table 1.

### 2.2. CT-Based Volumetric Assessment

**Figure 1:**
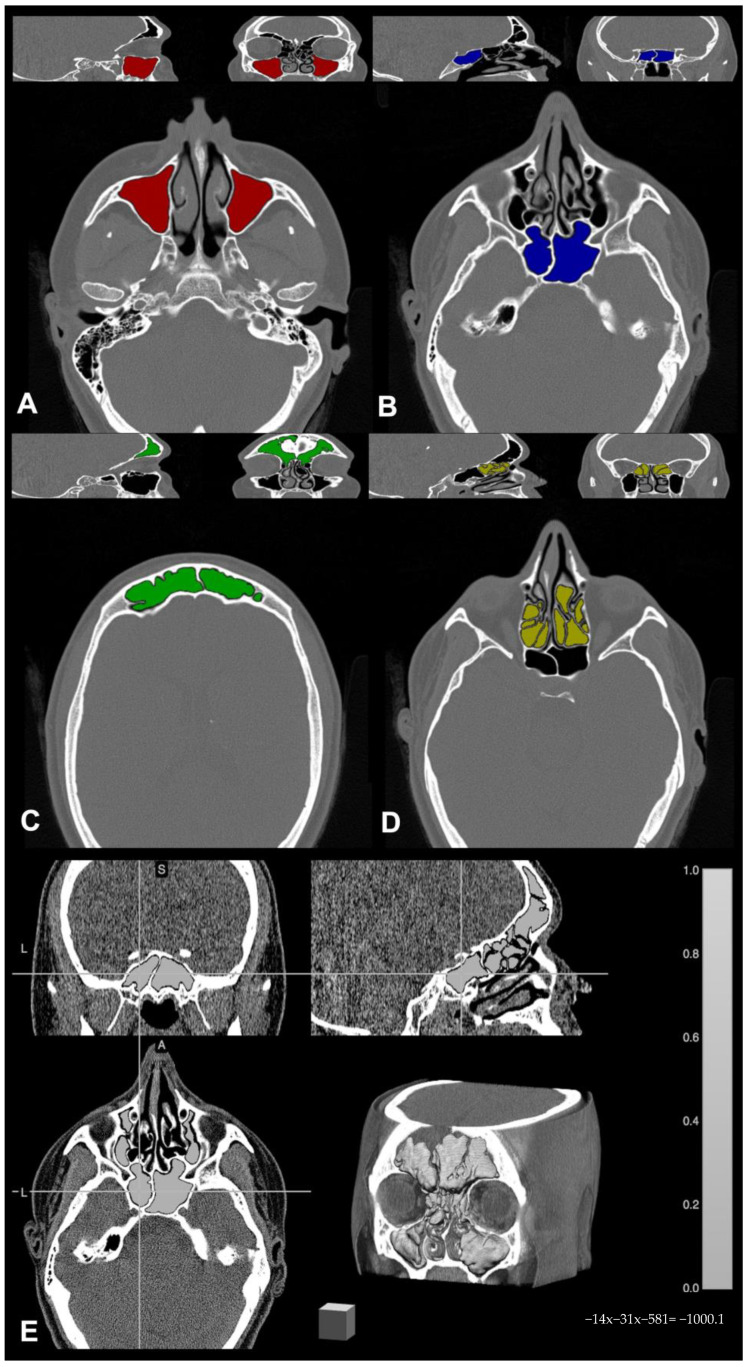
Example of paranasal sinus segmentation by means of MRIcron 2019 software (available at nitrc.org): (**A**) maxillary sinus (red); (**B**) sphenoid sinus (blue); (**C**) frontal sinus (green); (**D**) ethmoid sinus (yellow); (**E**) overall paranasal sinuses segmentation with three orthogonal plans and 3D reconstruction (light gray).

Paranasal sinus CT scans were stored and anonymized, then converted from DICOM to NIfTI format by using the free online tool dcm2niix (https://www.nitrc.org/plugins/mwiki/index.php/dcm2nii:MainPage, (accessed on 15 March 2020)). For each CT examination, paranasal sinuses were individually segmented by an experienced neuroradiologist, and masks were drawn on each scan using the freely available software MRIcron 2019 (https://www.nitrc.org/projects/mricron (accessed on 15 March 2020)). In detail, bony landmarks were identified to delineate the sinus on the axial CT scan slice that contained one or more cavities, and the area inside such boundaries was filled automatically using the specific tool in the same software; these manually segmented sinus cavities constituted the region of interest (ROI) binary masks for each sinus. In case of mucosal thickening or sinus occlusion, binary masks of occupancy in the ROIs were also generated with the same procedure, and ROI masks were subsequently combined with the occupancy masks to remove any boundary inconsistencies [26]. Once completed, sinus masks were independently saved in the volume of interest (VOI) file format, and these VOI masks were used for calculating the computational volumes; namely, the output concerning each sinus was saved in a report of volumetric descriptive data for further statistical analysis. An example of segmentation showing the four sinus volume masks superimposed to the relative native CT examination is shown in Figure 1.

### 2.3. Statistical Analysis

The comparability of CF and HC groups in terms of age and sex was preliminarily assessed using the *t*-test and chi-square test, respectively. Regarding age distribution, a t-test for two independent means showed a significant difference between the two groups (*t*-value is 5.19, *p*-value is <0.00001, thus significant at *p* < 0.05); however, when analyzing effect size measures to quantify the magnitude of difference between two groups, a medium Cohen’s *d* value of 0.6 was found. This means that the difference, although noticeable and statistically relevant, is not extremely large or meaningful; taking the clinical context into account, it was deemed that the moderate shift in age distribution between the populations does not significantly affect the interpretation of the results. Regarding sex distribution, a chi-square test of independence showed no significant difference between the two groups (chi-square statistic is 1.07; *p*-value is 0.3, thus not significant at *p* < 0.05).

The Kolmogorov–Smirnov test was applied to assess whether the distribution of the sample data conformed to a normal distribution. A two-tailed independent samples parametric *t*-test was used to compare the means of CF and HC groups in order to determine whether associated population means were significantly different in terms of sinus expansion. An original *p*-value less than 0.05 was set, then Bonferroni-corrected for multiple comparisons; for all analyses, a final Bonferroni-corrected significance level of *p* = 0.01 was set (0.05/5, as the number of comparisons performed). To measure how much the experimental group differs from the control group in terms of sinus expansion, effect size Hedges’ *g* was calculated. Finally, a *z*-score test for two population proportions was used to determine whether the two groups differ significantly in terms of major anatomical variants’ prevalence detected on the CT scans; the results were considered significant when the *p*-value was less than 0.05. Statistical analyses were performed using the XLStat^®^ v.2019 package (https://www.xlstat.com (accessed on 15 March 2020)).

## 3. Results

**Figure 2:**
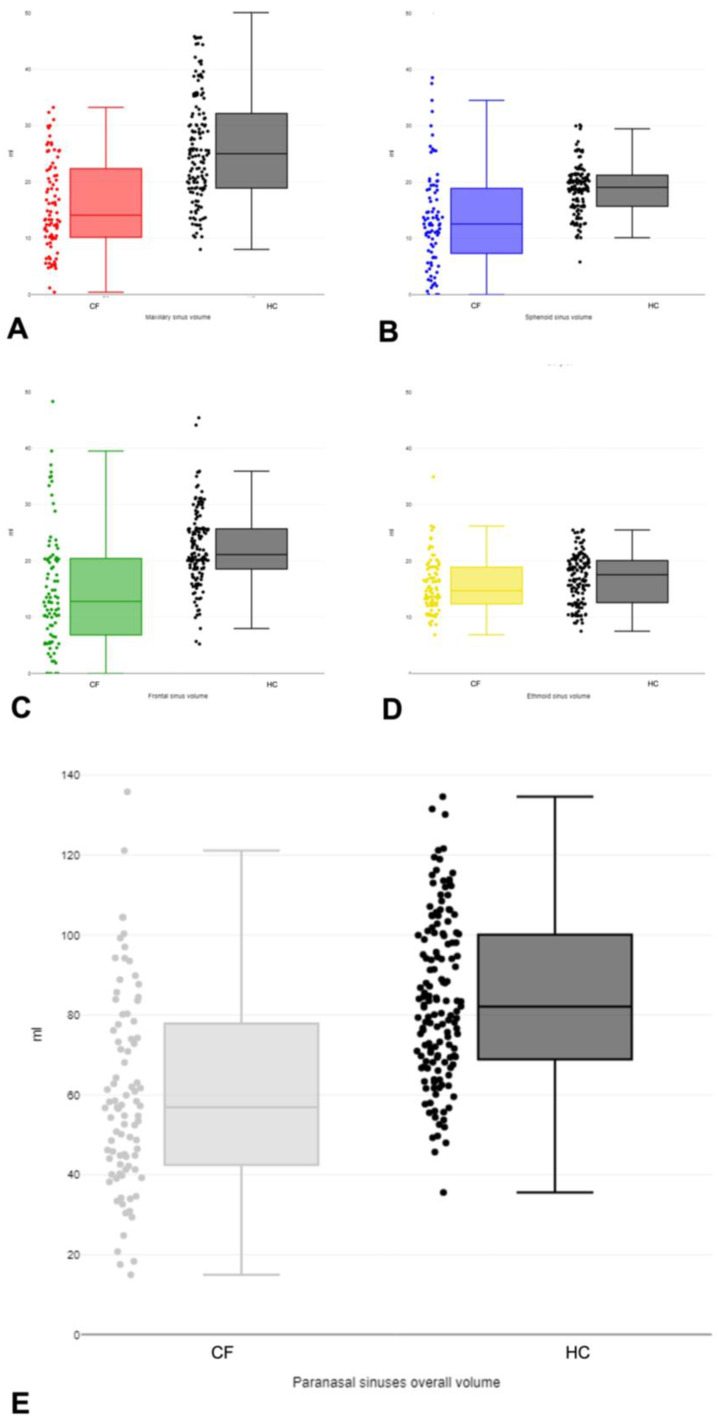
Scattered boxplots with data dispersion (on the left) and minimum/median/maximum values, quartiles, and interquartile ranges (on the right), representing paranasal sinus volumes in cystic fibrosis patients versus healthy controls (always plotted in dark gray): (**A**) maxillary sinus (red); (**B**) sphenoid sinus (blue); (**C**) frontal sinus (green); (**D**) ethmoid sinus (yellow); (**E**) overall paranasal sinuses (light grey). Volumes are expressed in mL.

The value of the Kolmogorov–Smirnov test statistic is D = 0.05057, with *p*-value = 0.57281; therefore, the data do not differ significantly from a normal distribution. The overall sinus volumes in the 89 CF participants compared to the 144 HCs were proved to differ significantly between the two groups (*p* < 0.00001); with the only exception of ethmoid sinus pneumatization, which was similar in both populations (*p* = 0.06), all the other sinuses were found to be statistically different. The maximum contribution in volume difference was from maxillary sinus (*p* < 0.00001, with effect magnitude *g* = 1.13), followed by frontal sinus (*p* = 0.000034, with effect magnitude *g* = 0.55), while sphenoid sinus (although with a statistically significant difference in volume between CF and HC) only provided a moderate contribution to the overall difference (*p* = 0.02, with effect magnitude *g* = 0.29, not significant after Bonferroni correction). A summary of sinus-specific volume results (reporting *t*-value, *p*-value, and Hedges’ *g* effect size) is shown in Table 2; descriptive statistics representing paranasal sinus volumes in CF patients versus HCs are visualized in Figure 2 as scattered boxplots, reporting data dispersion, minimum/median/maximum values, quartiles, and interquartile ranges.

Finally, a *z*-score test revealed no significant difference in terms of major anatomical variants between the two populations (i.e., for overall anatomical variants, *p*-value = 0.43, ranging from *p*-value = 0.11 for infraorbital canal dehiscence to *p*-value = 0.95 for sphenoid septum presence); a summary of sinus variants’ prevalence in the two groups (reporting *z*-score and *p*-value) is shown in Table 3.

## 4. Discussion

In this retrospective cohort study, the expansion of paranasal sinuses in adult CF patients evaluated by means of CT-based volumetric assessment is reduced, compared to HCs, with the only exception of ethmoid cells (and to a lesser extent, sphenoid sinus), whose volume is comparable in the two groups; the most significant volume reduction was observed for maxillary sinus, followed by frontal sinus and sphenoid sinus in this order (the latter not significant after correction for multiple comparisons). To the best of our knowledge, this is the first volumetric assessment of paranasal sinus pneumatization in adult CF. In line with previous evidence, our results provide new inferences on the impact of causative CF mutations on the development of paranasal sinuses and, therefore, on their final form and size.

From a developmental standpoint, paranasal sinuses appear as focal evaginations of the rhinonasal mucosa during fetal life, and then undergo acceleration in volume expansion after birth harmoniously with splanchnocranium growth and odontogenesis (from initial non-functional position up to permanent teeth eruption); ethmoid and maxillary sinuses are already partly visible in the newborn, whereas sphenoidal and frontal sinuses are not present at birth. Frontal and sphenoidal sinuses appear by the age of 2–4 y, mostly developing during secondary dentition and puberty; paranasal cavity expansion is almost complete by the age of 12 y when sinuses reach a size and form similar to an adult [27].

Abnormal paranasal sinus pneumatization is a common finding in CF. Frontal sinus aplasia, maxillary ethmoid and sphenoid sinus hypoplasia, and hypopneumatized anatomical variants of the paranasal sinuses have been commonly described, especially in younger CF patients with moderate-to-severe disease activity [12]. Indeed, several studies explored sinus expansion in the pediatric CF population, providing increasing evidence of reduced or lacking sinus pneumatization in children with CF [3,22]; among those studies, in a single recent paper, a CT-based volumetric analysis was performed to confirm such data and to determine how pervasive the alteration was during longitudinal age-related development of the paranasal sinuses [23,28]. Compared to this last study carried out on pediatric CF patients (aged < 18 y at the moment of CT examination), our results on adult CF patients (aged > 18 y at the moment of CT examination) are in line with the evidence of disease progression over time and of reduced pneumatization in older CF children compared to HCs; our findings were therefore consistent with the reported lower volume increase in maxillary sphenoidal and frontal sinuses in affected children compared to the control group, but we also implemented such observations by proving that ethmoidal cells were not as affected as the other cavities by the disease. An association between CF and impaired sinus development can therefore be envisaged, allowing one to speculate that CF significantly affects the growth of the paranasal sinuses in the adult population as well as in children. Mutations responsible for CF disrupt the normal age-related increase in sinus pneumatization and volume. This disruption is largely attributed to defective mucociliary clearance, which causes mucus to accumulate within the sinuses. It is plausible that this mucus retention hinders normal sinus expansion, compromises ventilation, and predisposes the sinuses to persistent inflammation. As a result, persistent mucus accumulation can play a key role in the abnormal development and under-distension of the sinuses.

Concerning the pattern of hypoexpansion documented in our CF sample, pneumatization was lacking in the maxillary > frontal > sphenoidal sinuses, in this order, with the only relevant exception of ethmoid cells. In our experience, ethmoid sinus is highly variable in the shape, inner structure, and quantity of air cells both in CF and HCs; however, its overall pneumatization volume is comparable in the two groups. This finding may be explained by considering the anatomical complexity and the embryological development of the ethmoid bone. On the one hand, regarding its anatomical complexity, the limited involvement of the ethmoid sinus complex may be attributed to its peculiar anatomical features and drainage patterns. Unlike other sinuses, the ethmoid sinuses drain through small ostia directly into the middle and superior meatus and maintain close anatomical relationships with both the frontal and maxillary sinuses, with drainage routes relatively complex but shorter. Conversely, the maxillary and frontal sinuses have longer and more convoluted drainage pathways, making them more dependent on gravity for effective mucus clearance [29]. Their greater dependence on gravity for drainage may make them more vulnerable to blockage, potentially hindering proper aeration and normal sinus development. On the other hand, regarding the development of the ethmoid, embryologically, ethmoid bone arises from ossification and primary pneumatization within the cartilaginous nasal capsule (so-called “intra-capsular” process); conversely, all the other sinuses originate from secondary pneumatization occurring along the lateral wall of the nasal capsule, where diverticular folds cross the borders of the nasal capsule and occupy air space in adjacent regions (so-called “extra-capsular” process) [30,31]. The ethmoid air space originates early from the nasal capsule and is therefore somehow constrained by the surrounding air spaces, while the size of the remaining sinuses (frontal, maxillary, and sphenoidal) arising “outside” the nasal capsule is more variable and less predictable. Therefore, we can speculate that ethmoid sinus relative sparing in CF patients compared to other sinuses may be explained by the strategic central role the nasal capsule and ethmoid bone play since intrauterine life in developmental trajectories of the nasal complex, and by the different expression of the CFTR gene along the fetal respiratory system [32].

Concerning the ancillary purpose of the paper, no difference emerged in the prevalence of major anatomical variants in the two groups, with the exception of dehiscence of the infraorbital canal, which was observed only in HCs. Although this finding does not reach the level of statistical significance (probably due to sample size), it is known that the descending infraorbital nerve is more frequent in cases of large maxillary sinus volume [33]; therefore, its actual prevalence in the CF population might be lower than in the general population.

This study has two main limitations. The retrospective design coupled with the lack of clinical/genetic correlation for CF patients does not allow for going beyond anatomical considerations, nor for speculating on the role of infection in sinuses’ reduced pneumatization or analyzing possible genotype–phenotype associations. Thus, the deep meaning of the presented volumetric findings should be interpreted with caution, and further validations incorporating clinical and genetic data are still warranted. Nevertheless, because of the relatively rare condition in adults (a still poorly known and explored area) and the use of computational volumetric CT-based tools for volume analysis, these findings confirming the overall reduction in bony sinus expansion in the CF adult population do offer interesting and potentially useful information on the disease. This is even more true considering that a deep understanding of sinus anatomy is pivotal for sinus surgery when functional endoscopic sinus surgery is envisaged (as is so often the case in the treatment of chronic rhinosinusitis of CF patients).

## Figures and Tables

**Table 1:** Cystic fibrosis patients and healthy controls’ demographics and anatomical variants. Legend: CF = cystic fibrosis; HC = healthy control; SD = standard deviation; CT = computed tomography.

		CF (n = 89)	HC (n = 144)
Age (years)	Mean	33.94	27.60
	SD	10.38	8.51
	Range	18–61	18–59
Sex	M	47	86
	F	42	58
Lund–Mackay CT score	Mean	7.71	0.90
	SD	5.20	1.77
Anatomical variants	Total (%)	29 (33%)	40 (28%)
	Concha bullosa (%)	4 (4%)	9 (6%)
	Infraorbital canal dehiscence (%)	0 (0%)	4 (3%)
	Sphenoid septum (%)	6 (7%)	10 (7%)
	Clinoid pneumatization (%)	1 (1%)	7 (5%)
	Onodi cell (%)	2 (2%)	4 (3%)
	Haller cells (%)	3 (3%)	3 (2%)
	Agger nasi (%)	2 (2%)	1 (1%)
	Ethmoid bulla (%)	2 (2%)	1 (1%)

**Table 2:** Summary of sinus-specific results reporting sinus volumes in the two groups (mean ± SD), *t*-value (Bonferroni-corrected), *p*-value, and Hedges’ *g* effect size measure. A *p*-value less than 0.05 is considered significant. Volumes are expressed in mL. * Not significant after Bonferroni correction.

	CF Sinus Volumes (mean ± SD)	HC Sinus Volumes (mean ± SD)	*t*-Value	*p*-Value	Hedges’ g
**Maxillary** **sinus**	15.82 ± 8.03	26.08 ± 9.59	−8.43	**<0.00001**	1.13
**Sphenoid** **sinus**	15.57 ± 16.03	18.81 ± 4.38	−2.23	0.02 *	0.29
**Frontal** **sinus**	16.41 ± 15.07	22.27 ± 6.76	−4.05	**0.000034**	0.55
**Ethmoid** **sinus**	15.69 ± 4.82	16.86 ± 4.33	−1.91	0.06	0.25
**Overall** **sinuses**	63.59 ± 35.12	83.88 ± 20.26	−5.59	**<0.00001**	0.75

**Table 3:** Summary of sinus variants’ prevalence in the two groups (reporting z-score and p-value). A *p*-value less than 0.05 is considered significant.

	CF	HC	z-Score	*p*-Value
**Total number**	29 (33%)	40 (28%)	0.78	0.43
**Concha bullosa**	4 (4%)	9 (6%)	−0.57	0.57
**Infraorbital canal dehiscence**	0 (0%)	4 (3%)	−1.59	0.11
**Sphenoid septum**	6 (7%)	10 (7%)	−0.06	0.95
**Clinoid pneumatization**	1 (1%)	7 (5%)	−1.52	0.13
**Onodi cell**	2 (2%)	4 (3%)	−0.25	0.8
**Haller cells**	3 (3%)	3 (2%)	0.6	0.55
**Agger nasi**	2 (2%)	1 (1%)	1.02	0.31
**Ethmoid bulla**	2 (2%)	1 (1%)	1.02	0.31

## Data Availability

The original contributions presented in this study are included in the article. Further inquiries can be directed to the corresponding author(s).

## Abbreviations

CF	cystic fibrosis
CFTR	cystic fibrosis transmembrane conductance regulator
CT	computed tomography
HC	healthy control
HU	Hounsfield unit
SD	standard deviation
ROI	region of interest
VOI	volume of interest
